# Base preference for inosine 3′-riboendonuclease activity of human endonuclease V: implications for cleavage of poly-A tails containing inosine

**DOI:** 10.1038/s41598-024-65814-7

**Published:** 2024-06-28

**Authors:** Kazuma Mitsuoka, Jung In Kim, Aya Yoshida, Akane Matsumoto, Narumi Aoki-Shioi, Shigenori Iwai, Isao Kuraoka

**Affiliations:** 1 Department of Chemistry, Faculty of Science, Fukuoka University, 8-19-1 Nanakuma, Jonan-ku, Fukuoka, 814-0180 Japan; 2 Graduate School of Engineering Science, Osaka University, 1-3 Machikaneyama, Toyonaka, Osaka 560-8531 Japan

**Keywords:** Deamination, Endonuclease V, Inosine, DNA repair, RNA editing, Poly-A tail, Biochemistry, Enzymes, RNA, Molecular biology, DNA damage and repair, RNA metabolism

## Abstract

Deamination of bases is a form of DNA damage that occurs spontaneously via the hydrolysis and nitrosation of living cells, generating hypoxanthine from adenine. *E. coli* endonuclease V (eEndoV) cleaves hypoxanthine-containing double-stranded DNA, whereas human endonuclease V (hEndoV) cleaves hypoxanthine-containing RNA; however, hEndoV in vivo function remains unclear. To date, hEndoV has only been examined using hypoxanthine, because it binds closely to the base located at the cleavage site. Here, we examined whether hEndoV cleaves other lesions (e.g., AP site, 6-methyladenine, xanthine) to reveal its function and whether 2′-nucleoside modification affects its cleavage activity. We observed that hEndoV is hypoxanthine-specific; its activity was the highest with 2′-OH modification in ribose. The cleavage activity of hEndoV was compared based on its base sequence. We observed that it has specificity for adenine located on the 3′-end of hypoxanthine at the cleavage site, both before and after cleavage. These data suggest that hEndoV recognizes and cleaves the inosine generated on the poly A tail to maintain RNA quality. Our results provide mechanistic insight into the role of hEndoV in vivo.

## Introduction

DNA damage can be caused by spontaneous deamination of bases through hydrolysis and nitrosation, leading to the substitution of adenine, cytosine, and guanine bases with hypoxanthine, uracil, and xanthine, respectively^1^. The substitution of deoxyinosine, a nucleoside formed when hypoxanthine is attached to a deoxyribose ring, occurs predominantly via two mechanisms: (1) spontaneous hydrolysis or nitrosation within the cell and (2) the insertion of dITP (a deaminated product of dATP) by DNA polymerase during replication, both necessitating repair to safeguard the genome. Consequently, deoxyinosine pairs with cytosine during replication because of its structural similarity to guanine, resulting in an A: T to G: C transition mutation^2,3^.

Two DNA repair pathways remove this mutagenic deoxyinosine in cells^4^: (a) the base excision repair (BER) pathway initiated by alkylidene DNA glycosylase (AAG)^5^, and (b) the alternative excision repair (AER) pathway^6^, which produces a single nick on one side of the damaged base and removes deoxyinosine using the exonuclease activity of DNA polymerase^7^. The AER pathway, involving *E. coli* endonuclease V (eEndoV), is biochemically distinct from the major DNA repair pathways, such as nucleotide excision repair (NER) and BER^8–11^. eEndoV recognizes double-stranded and single-stranded DNAs containing deoxyinosine, cleaving the second and third phosphodiester bonds at the 3′-end of deoxyinosine. Subsequent 3′- to 5′-exonuclease activity removes deoxyinosine, completing the DNA repair in the eEndoV-dependent AER pathway. Since deoxyinosine is an error-prone lesion, mutations in EndoV result in a strong mutator phenotype in *E. coli* and *S. pombe*^2,3,12^. However, the role of AER pathway in mammals remains unclear.

Deamination reactions may occur in RNA. Inosine is a nucleoside formed when hypoxanthine is attached to a ribose ring. Three fundamental mechanisms contribute to inosine production in RNA^4^: (a) DNA, spontaneous, and incidental modifications of inosine through deamination via hydrolytic and nitrosative reactions^1,13^; and (b) inosine triphosphate (ITP) is incorporated into the mRNA by RNA polymerase during transcription. Although ITP is removed from the cell nucleotide pool by inosine triphosphatase (ITPase), the incorporation of ITP is caused by an imbalance in purine nucleotide metabolism^14,15^, and (c) adenosine is converted to inosine by the RNA-editing enzymes, adenosine deaminases acting on RNA (ADAR) and tRNA (ADAT)^16–19^. The ADAR family comprises three members: ADAR1, ADAR2, and ADAR3. ADAR1 and ADAR2 catalyze RNA editing, whereas ADAR3 does not. ADAT converts adenosine into inosine in tRNA. Inosine is present at the wobble position of the anticodons.

Quantitative analyses of *E. coli* and *S. cerevisiae* revealed that the inosines in the RNA pools were present at background levels of 11 and 42 per 10^6^ nucleotides, respectively. These levels were higher than those of deoxyinosine in DNA (1.2 and 2.0 per 10^6^ nucleotides, respectively)^14,15^. Therefore, several inosine-containing RNAs are presumed to be present in living mammalian cells.

Previously, we analyzed the function of human endonuclease V (hEndoV) and confirmed that it cleaves DNA-containing deoxyinosines^20^. Because the amino acid sequences of eEndoV and hEndoV share 37% identity, we assumed that hEndoV could repair deoxyinosine-containing DNA. We observed that this enzyme can cleave RNA-containing inosines. A comparison of the cleavage activity of hEndoV on DNA and RNA showed that it preferentially cleaves inosine-containing RNA^20,21^. Therefore, hEndoV is an enzyme that removes RNA containing inosine but does not function in DNA repair^20^. Similarly, *Arabidopsis thaliana* endonuclease V is a ribonuclease specific for inosine-containing RNA^22^.

EndoV structures are similar in bacteria and mammals^23^. hEndoV interacts with inosine before and after the base at the cleavage site. eEndoV and *T. thermophilus* EndoV (tthEndoV) recognize DNA containing apurinic/apyrimidinic (AP) sites, urea base mismatches, and xanthines^8,24,25^. Unlike hEndoV, eEndoV, and tthEndoV recognize sites other than inosine. In this study, we investigated the following three factors to analyze the hEndoV function: (a) whether hEndoV cleaves RNA-containing inosines, (b) whether hEndoV needs a 2′-OH substitution in ribose, and (c) whether hEndoV prefers specific sequences. In this study, an inosine 3′ endonuclease activity of hEndoV revealed that hEndoV can cleave poly-A tails containing inosine. Our results suggested that hEndoV is involved in RNA metabolism.

## Results

### hEndoV is an inosine-specific ribonuclease

EndoV homologs have highly conserved amino acid sequences and structures. eEndoV and tthEndoV cleave DNA containing AP sites, urea base mismatches, or xanthines (Fig. S1A; Table 1). To investigate whether hEndoV could cleave lesions other than deoxyinosine, we tested the cleavage activity of hEndoV on substrates (Fig. S1A), containing deoxyinosine (Fig. S1B, lanes 1–4), and AP sites (Fig. S1B, lanes 5–8), and 6-methyladenine (Fig. S1B, lanes 9–12), and xanthine produced by the deamination of guanine (Fig. S1B, lanes 13–16). Once the oligonucleotides containing deoxyinosine were cleaved by hEndoV (Fig. S1B, lanes 1–4, Fig. S1C), nuclease activity of hEndoV was not observed (Fig. S1B, lanes 5–16, Fig. S1C).

**Table 1:** DNA and RNA oligos for human EndoV nuclease activity assay (Related to Figs. 1–5 and Figures S1–S3).

Nuclease activity assay of human EndoV for inosine							
Name	Sequence						
ssDNA containing deoxyinosine	5'-CTGTATGAT	G	dI	rA	G	A	TGCTGAC-3'
ssDNA containing AP site	5'-CTGTATGAT	G	AP	rA	G	A	TGCTGAC-3'
ssDNA containing 6-methyladenine	5'-CTGTATGAT	G	Me-A	rA	G	A	TGCTGAC -3'
ssDNA containing xanthine	5'-CTGTATGAT	G	X	rA	G	A	TGCTGAC-3'

Nuclease activity assay of human EndoV for ribo-adenine							
Name	Sequence						
dI next to ribo-A	5'-CTGTATGAT	G	dI	rA	G	A	TGCTGAC -3'
dI next to deoxy-A	5'-CTGTATGAT	G	dI	dA	G	A	TGCTGAC -3'
dI next to 2'-O-Me-A	5'-CTGTATGAT	G	dI	O-Me-A	G	A	TGCTGAC -3'
dI next to 2'-F-A	5'-CTGTATGAT	G	dI	F-A	G	A	TGCTGAC -3'

Nuclease activity assay of human EndoV for nucleotide located -1 of cleavage site							
Name	Sequence						
dI next to ribo-A	5'-CTGTATGAT	G	dI	rA	G	A	TGCTGAC -3'
dI next to ribo-C	5'-CTGTATGAT	G	dI	rC	G	A	TGCTGAC -3'
dI next to ribo-G	5'-CTGTATGAT	G	dI	rG	G	A	TGCTGAC -3'
dI next to riboU	5'-CTGTATGAT	G	dI	rU	G	A	TGCTGAC -3'

Nuclease activity assay of human EndoV for nucleotide located + 1 cleavage site							
Name	Sequence						
dI two next to ribo-A	5'-CTGTATGAT	G	dI	rA	rA	A	TGCTGAC-3'
dI two next to ribo-C	5'-CTGTATGAT	G	dI	rA	rC	A	TGCTGAC-3'
dI two next to ribo-G	5'-CTGTATGAT	G	dI	rA	rG	A	TGCTGAC-3'
dI two next to ribo-U	5'-CTGTATGAT	G	dI	rA	rU	A	TGCTGAC-3'

Nuclease activity assay of human EndoV for nucleotide located + 2 of cleavage site							
Name	Sequence						
dI third next to ribo-A	5'-CTGTATGAT	G	dI	rA	rA	rA	TGCTGAC-3'
dI third next to ribo-C	5'-CTGTATGAT	G	dI	rA	rA	rC	TGCTGAC-3'
dI third next to ribo-G	5'-CTGTATGAT	G	dI	rA	rA	rG	TGCTGAC-3'
dI third next to ribo-U	5'-CTGTATGAT	G	dI	rA	rA	rU	TGCTGAC-3'

Nuclease activity assay of human EndoV for nucleotide located -3 of cleavage site							
Name	Sequence						
dI ahead of ribo-A	5'-CTGTATGAT	rA	dI	A	G	A	TGCTGAC-3'
dI ahead of ribo-C	5'-CTGTATGAT	rC	dI	A	G	A	TGCTGAC-3'
dI ahead of ribo-G	5'-CTGTATGAT	rG	dI	A	G	A	TGCTGAC-3'
dI ahead of ribo-U	5'-CTGTATGAT	rU	dI	A	G	A	TGCTGAC-3'

Nuclease activity assay of human EndoV for ribo-adenine							
Name	Sequence						
dl next to rA dA dA	5'-CTGTATGAT	G	dI	rA	dA	dA	TGCTGAC-3'
dl next to rA rA dA	5'-CTGTATGAT	G	dI	rA	rA	dA	TGCTGAC-3'
dl next to rA dA dA	5'-CTGTATGAT	G	dI	rA	rA	rA	TGCTGAC-3'

Nuclease activity assay of human EndoV for poly-A							
Name	Sequence						
Poly-A	5'-rArArArArArArArArArArArArArArArArArArArArA- 3'						
i-PolyA	5'-rArArArArArArArArArArIrArArArArArArArArArA-3'						

## Effect of hEndoV cleavage activity on qualified 2′ of nucleoside

eEndoV cleaves ssDNA-containing deoxyinosines more specifically than ssRNA-containing deoxyinosines. On the contrary, hEndoV preferred ssRNA containing inosine over ssDNA containing deoxyinosine and a hydroxy substitution in ribose located at 3′ to inosine^20^. Thus, we assumed that hydroxy in the 2′ position in nucleoside affects the cleavage activity of hEndoV compared to DNA and RNA.

To confirm whether substitution at 2′ of nucleoside affects the cleavage activity of hEndoV, we tested the nuclease activity of hEndoV on single-strand oligonucleotides containing ribo (hydroxy), deoxy, methoxy, and fluorine substitutions (Fig. S2A; Table 1). We observed that hEndoV preferred hydroxy substitution at 2′ in nucleoside (Fig. S2B, lanes 1–4, Fig. S2C) compared to deoxy, methoxy, and fluorine substitutions (Fig. S2B, lanes 5–16, Fig. S2C). In other words, this protein shows high RNA cleavage activity. Our results suggest that substituting nucleoside at 2′ plays a key role in the cleavage activity of hEndoV.

## The cleavage activity of hEndoV depends on the nucleotide sequence

Previous studies on the crystal structure of EndoV and a comparison of the alignment of the amino acid sequences of EndoV homologs revealed the following characteristic similarities between hEndoV and eEndoV: identity = 74/199 (37%), positivity = 108/199 (54%), and gap = 19/199 (10%)^20^, demonstrating that these proteins are highly conserved in bacteria and humans. Notably, electrophoretic mobility shift assays revealed that hEndoV has affinity for both RNA- and DNA-containing inosines. This enzyme also has an affinity for dsRNA containing no inosine, indicating that the inosine in RNA is dispensable for RNA binding of hEndoV^20^.

However, our structural analyses revealed that EndoV binds closely to the 1 and + 1 bases of the cleavage site^23^. Therefore, we investigated whether the base sequence of the deoxyinosine affected the cleavage activity of hEndoV.

**Figure 1:**
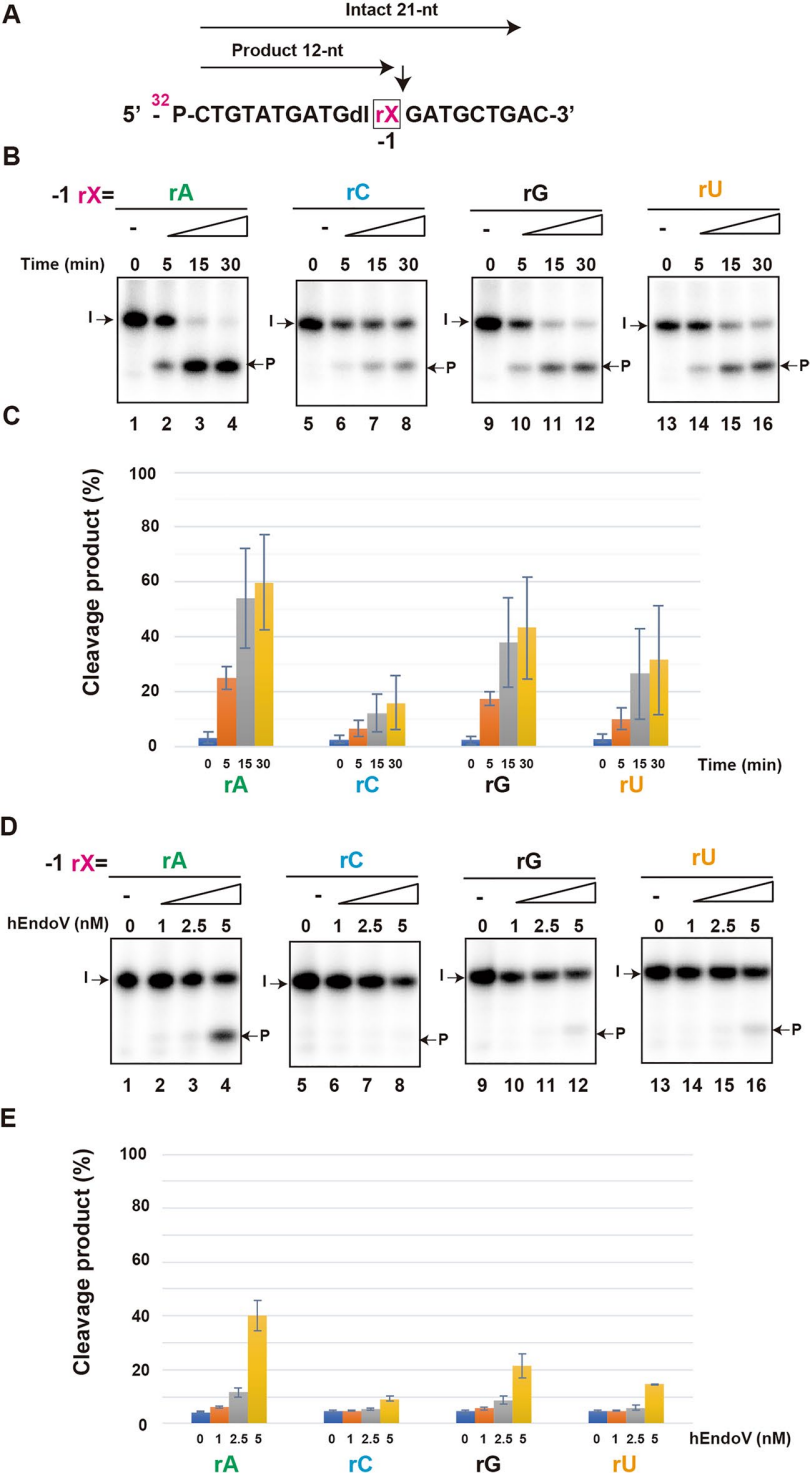
hEndoV prefers adenine base located at the –1 position site. (**A**) ^32^P-labeled substrates containing rX (rA, rC, rG, or rU) at the –1 position are indicated. Arrows indicate the position of cleavage. Intact 21-nt; Intact oligonucleotide. Product 12-nt; cleavage products. (**B**) The ^32^P-labeled substrate containing rA (lanes 1–4), rC (lanes 5–8), rG (lanes 9–12), and rU (lanes 13–16) were incubated with 5 nM hEndoV at 37 ℃ for indicated time. The cleavage products were analyzed by denaturing 12.5% urea gel electrophoresis. The arrows mark the positions of intact oligonucleotide (I), and product (P) after hEndoV endonuclease action. (**C**) Graphs showing the yield of cleavage products obtained from (**B**) hEndoV cleavage activity. (**D**) The ^32^P-labeled substrate containing rA (lanes 1–4), rC (lanes 5–8), rG (lanes 9–12), and rU (lanes 13–16) were incubated with indicated concentrations of hEndoV for 30 min. (**E**) Graphs showing the yield of cleavage products obtained from (**D**) hEndoV cleavage activity. Abbreviations: hEndoV, human endonuclease V; P, phosphorus; rA, riboadenine; rC, ribocytosine; rG, riboguanine; rU, ribouracil. Representatives of three independent experiments are shown.

First, we compared the cleavage activity by changing the base located next to the 3′ of deoxyinosine, replacing the –1 located base of the cleavage site with adenosine (rA), cytidine (rC), guanosine (rG), and uridine (rU) (Fig. 1A; Table 1). For statistical analysis, time-course and concentration experiments were performed thrice (Fig. 1B,D), and the cleavage products were quantified (Fig. 1C,E). We observed that the cleavage activity of hEndoV was the highest with the adenine base in a time- and concentration-dependent manner (Fig. 1B,D, lanes 1–4, Fig. 1C,E).

**Figure 2:**
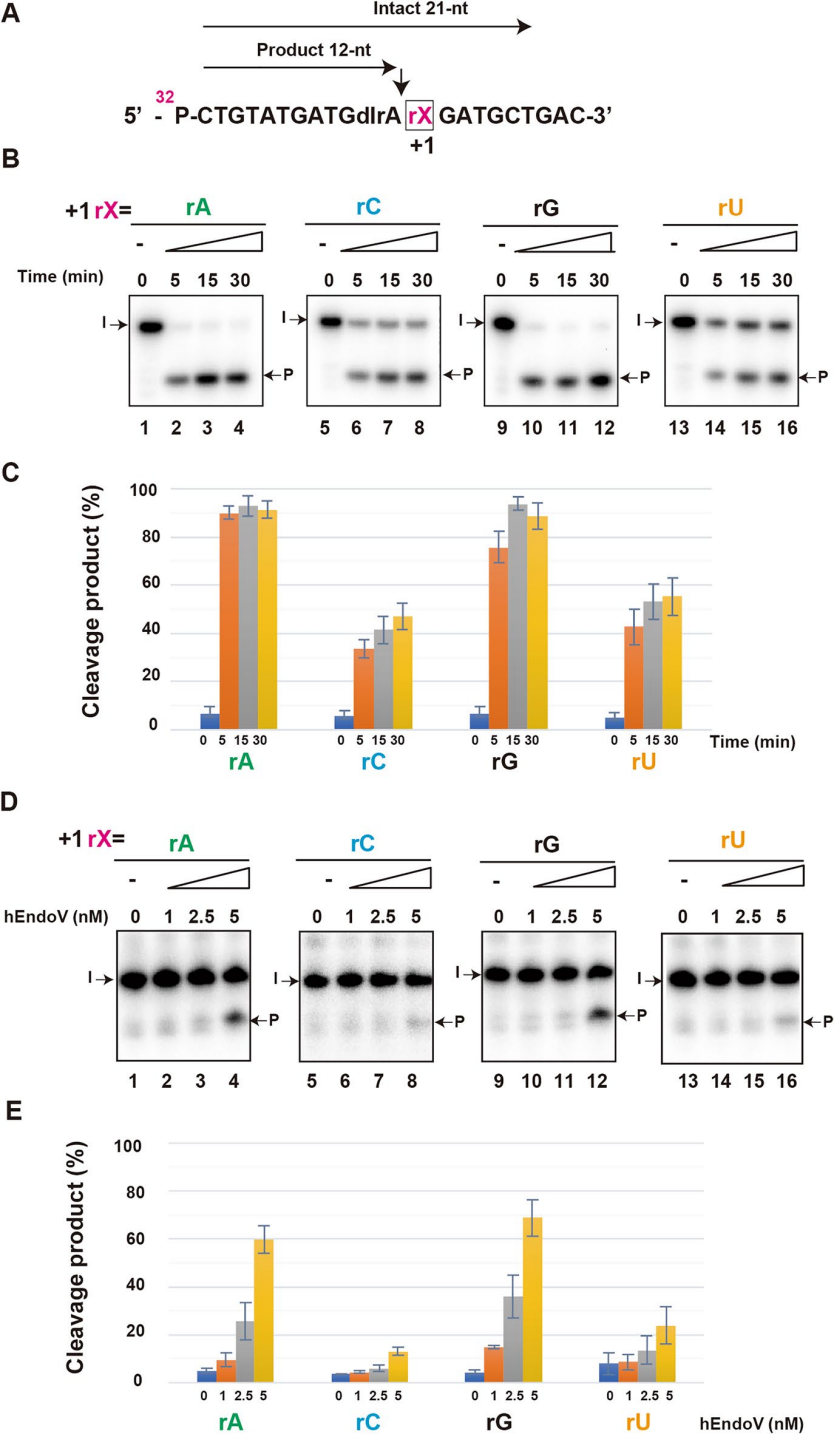
hEndoV prefers adenine base located at the + 1 position site. (**A**) ^32^P-labeled substrates containing rX (rA, rC, rG, or rU) at the + 1 position. Arrows indicate cleavage positions. Intact 21-nt; Intact oligonucleotide. Product 12-nt; cleavage products. (**B**) The ^32^P-labeled substrate containing rA (lanes 1–4), rC (lanes 5–8), rG (lanes 9–12), and rU (lanes 13–16) were incubated with 5 nM hEndoV at 37 ℃ for indicated time. The cleavage products were analyzed by denaturing 12.5% urea gel electrophoresis. Arrows mark the positions of the intact oligonucleotide (I) and the product (P) after hEndoV endonuclease activity. (**C**) Graphs showing the yield of the cleavage products obtained from (B) hEndoV cleavage. (**D**) ^32^P-labeled substrates containing rA (lanes 1–4), rC (lanes 5–8), rG (lanes 9–12), and rU (lanes 13–16) were incubated with the indicated concentrations of hEndoV for 30 min. (**E**) Graphs showing the yield of cleavage products obtained from (**D**) hEndoV cleavage. Abbreviations: hEndoV, human endonuclease V; P, phosphorus; rA, riboadenine; rC, ribocytosine; rG, riboguanine; rU, ribouracil Representatives of three independent experiments are shown.

Next, we changed the second base located at 3′ of inosine, replacing the + 1 located base of the cleavage site with rA, rC, rG, and rU to compare the cleavage activity of hEndoV (Fig. 2A; Table 1). The cleavage activity of hEndoV with rA and rG was higher than that with rC and rU in a time- and concentration-dependent manner (Fig. 2B,E, lanes 1–4 and lanes 9–12, Fig. 2C,E). These results suggest that the –1 and + 1 bases, to which hEndoV binds, are important for its cleavage activity and that hEndoV has specificity for rA.

**Figure 3:**
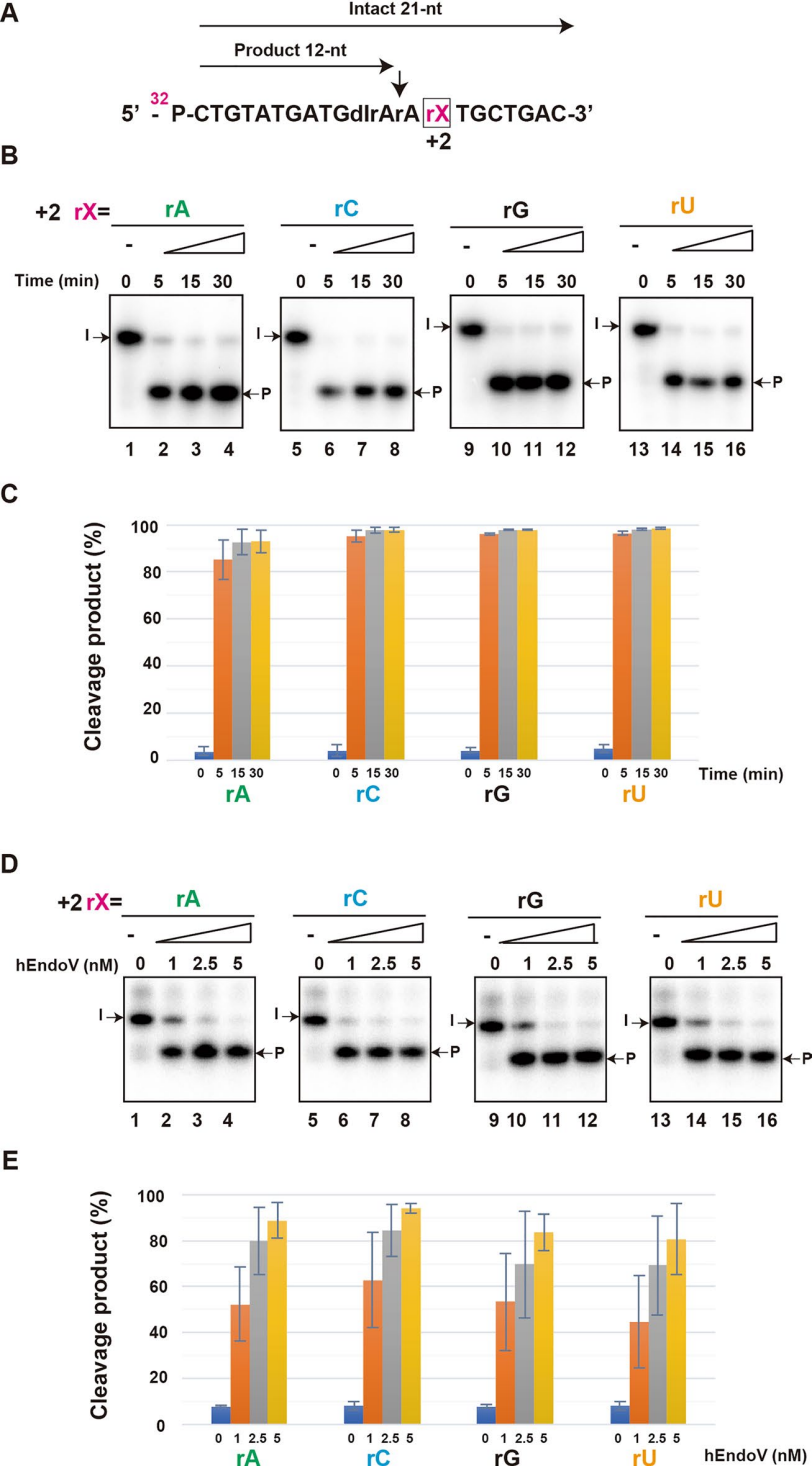
hEndoV prefers adenine base located at the + 2 position site. (**A**) ^32^P-labeled substrates containing rX (rA, rC, rG, or rU) at the + 2 position. Arrows indicate cleavage positions. Intact 21-nt; Intact oligonucleotide. Product 12-nt; cleavage products. (**B**) The ^32^P-labeled substrate containing rA (lanes 1–4), rC (lanes 5–8), rG (lanes 9–12), and rU (lanes 13–16) were incubated with 5 nM hEndoV at 37 ℃ for indicated time. The cleavage products were analyzed by denaturing 12.5% urea gel electrophoresis. Arrows mark the positions of the intact oligonucleotide (I) and the product (P) after hEndoV endonuclease activity. (**C**) Graphs showing the yield of the cleavage products obtained from (B) hEndoV cleavage. (**D**) ^32^P-labeled substrates containing rA (lanes 1–4), rC (lanes 5–8), rG (lanes 9–12), and rU (lanes 13–16) were incubated with the indicated concentrations of hEndoV for 30 min. (**E**) Graphs showing the yield of cleavage products obtained from (**D**) hEndoV cleavage. Abbreviations: hEndoV, human endonuclease V; P, phosphorus; rA, riboadenine; rC, ribocytosine; rG, riboguanine; rU, ribouracil Representatives of three independent experiments are shown.

Finally, we changed the third base located at 3′ of inosine (+ 2 located base of cleavage site), and the base located at 5′ to inosine (–3 located base of cleavage site), replacing them with rA, rC, rG, and rU to compare the cleavage activity of hEndoV (Fig. 3A and Fig. S3A; Table 1).

However, we observed no difference in the cleavage activity of hEndoV, regardless of the type of replacement at the + 2 located base (Fig. 3B–E) in a time- and concentration-dependent manner or at the 3 located base (Fig. S3), in a time-dependent manner. Based on these results, we suggest that the cleavage activity of hEndoV is specific to the adenine base located at the cleavage site, implying that hEndoV prefers the IAA base sequence.

## hEndoV prefers a hydroxy substitution in adenosine at + 1 position

**Figure 4:**
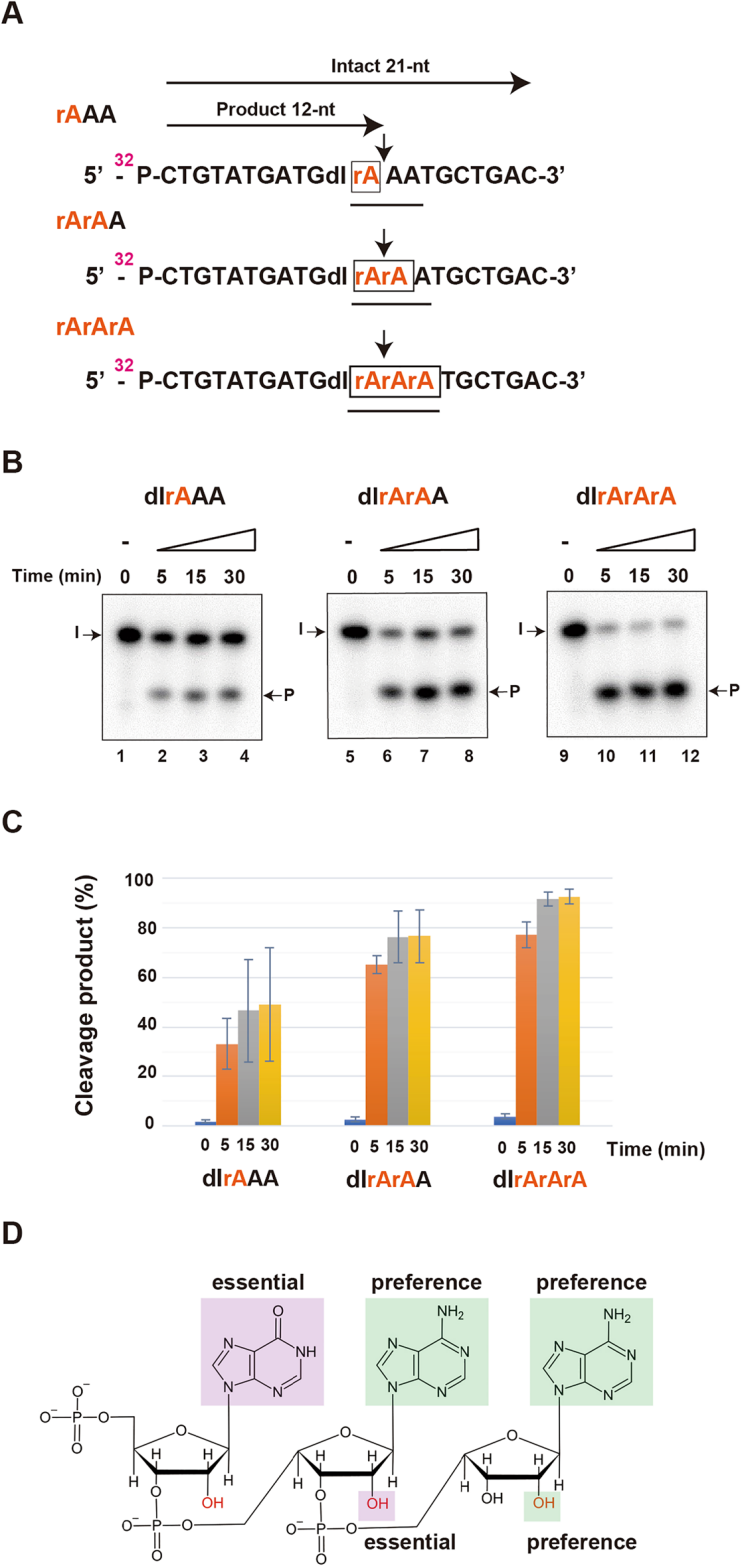
hEndoV prefers a hydroxy (–OH) modification of adenosine. (**A**) ^32^P-labeled substrates containing deoxyinosine and a series of adenosine modifications are shown. Intact 21-nt; Intact oligonucleotide. Product 12-nt; cleavage products. (**B**) ^32^P-labeled substrate containing deoxyinosine and rAAA (lanes 1–4), rArAA (lanes 5–8) and rArArA (lanes 9–12) were incubated with 5 nM hEndoV at 37 ℃ for 0, 5, 15 and 30 min. (**C**) The cleavage products were analyzed by denaturing 12.5% urea gel electrophorese. (**D**) Preference of chemical structure in hEndoV cleavage activity. Abbreviations: hEndoV, human endonuclease V; P, phosphorus; rA, ribo-adenine; dI, deoxyinosine; A, deoxy-adenine Representatives of three independent experiments are shown.

A previous study showed that hEndoV recognizes both ssDNA and dsDNA containing deoxyinosine, and ssRNA and dsRNA containing inosine. Additionally, we confirmed that this protein exhibits nuclease activity against RNA. Only slight cleavage activity was observed for ssDNA, albeit only slightly^26^. Therefore, we compared the cleavage activity of hEndoV for 2′-OH substituted ribose by changing the – 1, + 1, and + 2 located base sequences to deoxyadenosine (A) and adenosine (rA) to confirm whether hEndoV requires a hydroxy substitution at 2′ of ribose and an adenine base (Fig. 4A; Table 1). Cleavage activity was slightly lower for the rAAA sequence and approximately the same for the rArAA and rArArA sequences. These results suggest that the OH group in adenosine at the + 1 position contributes to activity (Fig. 4B,C). These results confirmed that hEndoV prefers the IAA sequence (Fig. 4D).

## hEndoV can cleave poly-A tail containing inosine

**Figure 5:**
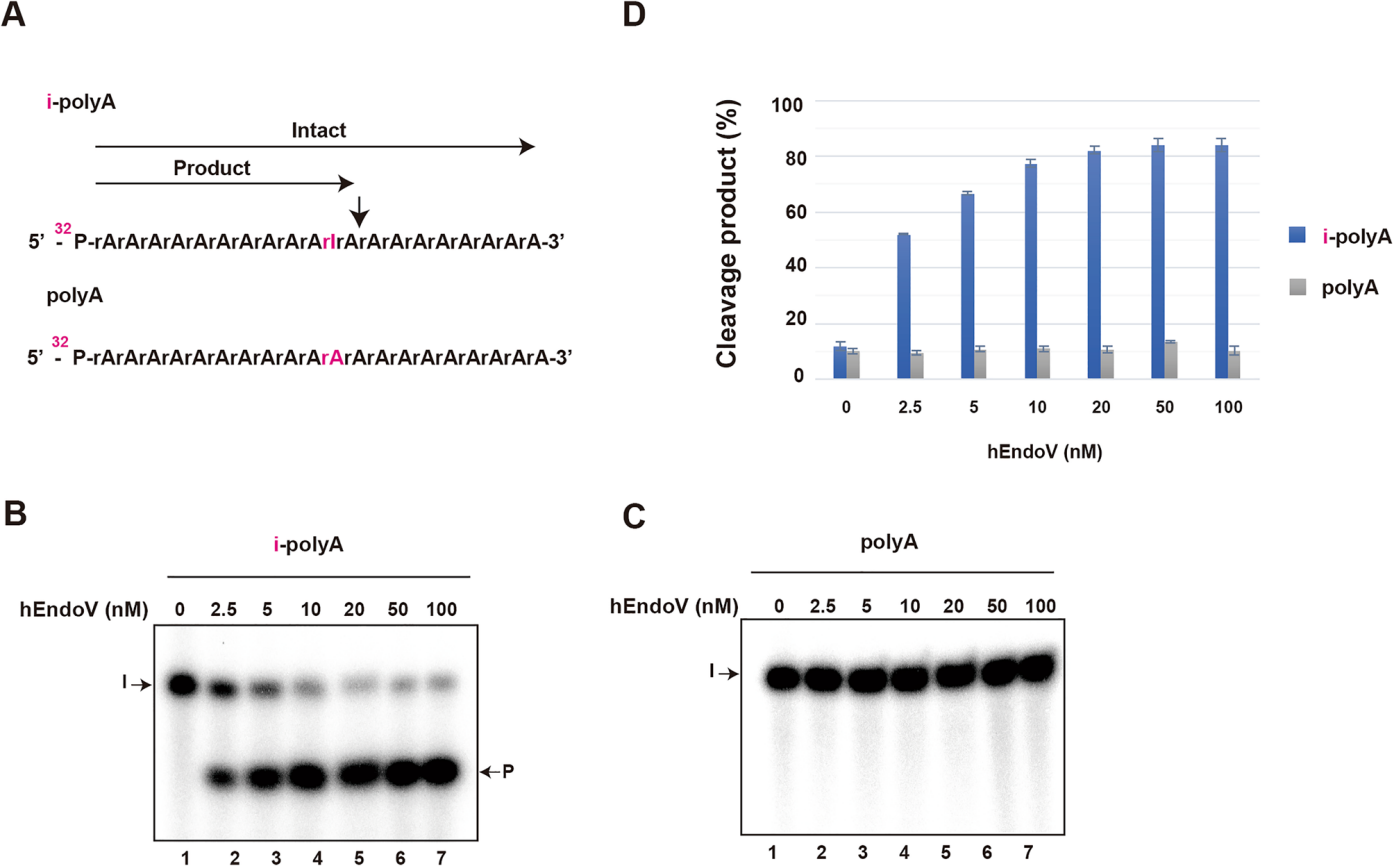
hEndoV can cleave poly-A tail containing inosine. (**A**) ^32^P-labeled inosine-containing poly-A (i-poly-A) or poly-A substrates are shown. ^32^P-labeled (**B**) i-poly-A substrates and (**C**) poly-A were incubated with indicated concentrations of hEndoV for 30 min. (**D**) The cleavage products were analyzed by denaturing 12.5% urea gel electrophorese. Abbreviations: hEndoV, human endonuclease V; P, phosphorus; rA, ribo-adenine; rI, inosine; i-polyA, inosine-containing poly-A; polyA, poly-A Representatives of three independent experiments are shown.

As our data indicate that hEndoV prefers ssRNAs containing inosine and adenine, which are located at the cleavage site before and after cleavage, we hypothesized that hEndoV recognizes and cleaves the poly (A) tail of RNA, including inosine. To confirm this, we examined whether hEndoV cleaves the poly A tail containing inosine using 21-mer-labeled poly A tail substrates and poly A tail containing inosine (i-poly A tail substrate) (Fig. 5A; Table 1). As expected, the results showed that hEndoV cleaved the i-poly-A tail in a concentration-dependent manner, but not the poly-A tail substrates (Fig. 5B,C,D).

## Discussion

In the present study, we analyzed the cleavage activity of hEndoV. Although hEndoV is structurally similar to eEndoV, which recognizes AP sites and mismatched bases, it shows high specificity only for inosines^8,24,25^. We examined whether hEndoV can cleave substrates other than RNA and observed that the cleavage activity was higher in the presence of a 2′ modification in ribose sugar. The cleavage activity of hEndoV was compared based on alterations in the base sequences. hEndoV showed the highest preference for adenine residues and its cleavage activity was highest when the bases before and after the cleavage site were adenine residues. In addition, cleavage activity was higher when the bases before and after the cleavage site contained ribose rather than deoxyribose, indicating that hEndoV prefers RNA to DNA. Taken together, we showed that hEndoV has endonuclease activity, which specifically cleaves single-stranded RNA containing inosine; the cleavage activity tends to be higher when the base on the 3′ end of inosine is adenine.

## Intracellular functions of hEndoV

Studies have shown that eEndoV efficiently cleaves DNA-containing deoxyinosine and acts as a DNA repair enzyme in the AER pathway^9,10,27^. In contrast, hEndoV is less active on deoxyinosine-containing DNA and preferentially cleaves inosine-containing RNA. It has been reported that hEndoV is localized in the cytoplasm^20^. Furthermore, human alkyladenine DNA glycosylase (hAAG) in the BER pathway is a DNA glycosylase responsible for recognizing deoxyinosine^28,29^. Therefore, hEndoV is believed to remove RNAs with inosine rather than repair deoxyinosine-damaged DNAs. Our data suggests that hEndoV preferred inosine-containing RNA substrates with the 5′-IAA-3′ sequence and could cleave an inosine-containing poly-A RNA substrate. Therefore, we hypothesized that hEndoV functions against the inosine generated in the poly A tail of RNA and removes untranslatable mature mRNAs containing inosine due to RNA damage.

The poly A tail is a significant modification in RNA processing and is involved in RNA stability and translation efficiency^30,31^. Polyadenylation involves the addition of up to 250 adenosine residues by poly A polymerase. The 5′-capping structure in mature mRNA is directly bound by the translation factors with the poly-A binding protein (PABP), thus forming a complex that circularizes the mRNA^32,33^. When inosine is introduced into the poly A tail, it is recognized as guanine, and PABP barely binds to the poly A tail, forming an unstable mRNA ring.

We believe that inosine is generated in the poly (A) tail through three main mechanisms. First is incorporating ITP by poly-A polymerase, which recognizes the 2- carbon of ATP and uses only ATP as a substrate. This enzyme cannot incorporate guanosine triphosphate (GTP) in which the 2′- carbon is present in an amino group^14,18^. However, it may be able to incorporate ITP, the deaminated product of ATP, because 2′- carbon in ITP has the same structure as that in ATP.

The second method is RNA editing using ADARs^16–18^. These enzymes convert adenosine residues into inosines in double-stranded RNA but not in single-stranded RNA. Because poly (A) tails are single-stranded RNA, ADARs may barely produce inosine.

The third is spontaneous deamination by hydrolysis or nitrosation reactions^1,13^. Chemical reactions are generally reliable in living cells. As these deamination reactions occur automatically in a time-dependent manner, the adenosine in the poly A tail of the mRNA may be partially converted to inosine. We believe that hEndoV contributes to the removal of mature mRNA by recognizing and cleaving the inosine generated on the poly A tails for RNA quality control.

## Materials and methods

### Proteins

The hEndoV protein was expressed in *E. coli* Rosetta 2 (Novagen) using pGEX-hEndoV and purified using DEAE Sepharose FF, Glutathione Sepharose 4 Fast Flow, and HiTrap Heparin HP columns (GE Healthcare) following the manufacturer's instructions. The proteins from the HiTrap Heparin HP column were eluted using a solution containing 300 mM KCl, 20 mM Tris–HCl buffer (pH 8.0), 10% glycerol, 0.1 mM ethylenediaminetetraacetic acid, and 1 mM dithiothreitol. Protein concentrations were measured using the Bio-Rad Protein Assay Kit (Bio-Rad, Hercules, CA, USA). hEndoV was observed as a single band on SDS-PAGE with Coomassie blue staining, according to a previous report^20^. The T4 polynucleotide kinase was obtained from New England Biolabs (Ipswich, MA, USA).

## DNA and RNA oligonucleotides

The DNA and RNA oligonucleotides used in this study are listed in Table 1. These oligonucleotides were synthesized at FASMAC (Kanagawa, Japan) and purified using high-performance liquid chromatography (HPLC). For the nuclease assay, oligonucleotides were phosphorylated at 5′ position using (γ-32P)-ATP and T4 polynucleotide kinase. The unincorporated nucleotides were removed using a MicroSpin G-25 column (Cytiva, Marlborough, MA, USA). Because the nuclease activity of hEndoV is unchanged between inosine and deoxyinosine and requires a second ribose at 3′ to the lesion^20^, here we used a DNA substrate containing the second ribose at 3′ to the deoxyinosine (Table 1).

## hEndoV nuclease assays

Reaction mixtures (5 μL) containing 0.2 nM of ^32^P-labelled oligonucleotides and the indicated amount of hEndoV were added in a reaction buffer containing 50 mM potassium acetate, 20 mM Tris–acetate, 10 mM magnesium acetate, and 1 mM dithiothreitol (DTT) (pH 7.9). The mixtures were incubated at 37 ℃ for the indicated time or concentration and then added to sequencing stop buffer (5 μL) containing 98% formamide, 20 mM ethylenediaminetetraacetic acid (EDTA), 0.5% bromophenol blue, and 0.5% xylene cyanol to terminate the reaction. The reaction products were separated on 12.5% denaturing urea gels. The dried gel was analyzed using a Fuji FLA-7000 phosphorimeter (Fujifilm, Tokyo, Japan). The products were quantified using the Fuji MultiGauge Software (Fujifilm, Tokyo, Japan). Photostimulated luminescence (*PSL*) was used for product counting. Cleavage products (%) = (products / (products + intact oligonucleotides)) × 100. The same experiment was performed thrice for statistical analysis. The error bars represent the standard error of the mean (SEM).

### Supplementary Information

Supplementary Figures.

## Data Availability

All main data of the study appear in the submitted article. Supplementary data are available online.
