# Supplementary material for: Base preference for inosine 3′-riboendonuclease activity of human endonuclease V: implications for cleavage of poly-A tails containing inosine
Source: Sci Rep. 2024 Jun 28;14:14973. doi: 10.1038/s41598-024-65814-7 (PMC11217400; doi:10.1038/s41598-024-65814-7)
Supplement: Supplementary file 1 — Supplementary Figures. [file 41598_2024_65814_MOESM1_ESM.docx]

**Supplementary Information 1:**

**Base preference for inosine 3′-riboendonuclease activity of human endonuclease V: implications for cleavage of poly-A tails containing inosine**

Kazuma Mitsuoka^1^, Jung In Kim^2^, Aya Yoshida^1^, Akane Matsumoto^1^, Narumi Aoki Shioi^1^, Shigenori Iwai^2^, Isao Kuraoka^1*^

^1^ Department of Chemistry, Faculty of Science, Fukuoka University, 8-19-1 Nanakuma, Jonan-ku, Fukuoka 814-0180, Japan

^2^ Graduate School of Engineering Science, Osaka University, 1-3 Machikaneyama, Toyonaka, Osaka 560-8531, Japan

***Correspondence:** Isao Kuraoka (kuraoka@fukuoka-u.ac.jp): ORCID iD https://orcid.org/0000-0001-6391-3411

**Figure S1. hEndoV cleaves deoxyinosine-containing substrates**

(A) ^32^P-labelled 21nt ssDNA containing deoxyinosine; an AP site, 6-methyladenine, or xanthine was used as the substrate. Arrows indicate the position of the cleavage. Intact 21-nt; Intact oligonucleotide. Product 12-nt; cleavage products. (B) ^32^p-labeled 21nt ssDNA containing deoxyinosine (lanes 1–4), AP site (lanes 5–8), 6-methyladenine (lanes 9–12), and xanthine (lanes 13–16) were incubated with 5 nM hEndoⅤ at 37 ℃ for 0, 5, 15 and 30 min. The cleavage products were analyzed by denaturing 12.5% urea gel electrophoresis. The chemical structures of inosine, the AP site, 6-methyladenine, and xanthine are shown in the upper panel. The arrows mark the positions of intact oligonucleotide (I), and product (P) after hEndoV endonuclease action. (C) Graph showing the yield of cleavage products obtained by hEndoV cleavage activity. The products were quantified using a ImageJ Software. Error bars represent Standard Deviation (SD). Abbreviations: hEndoV, human endonuclease V; P, phosphorus; dI, deoxyinosine; AP, apurinic/apyrimidinic; mA, 6-methyladenine; dX, xanthine.

**
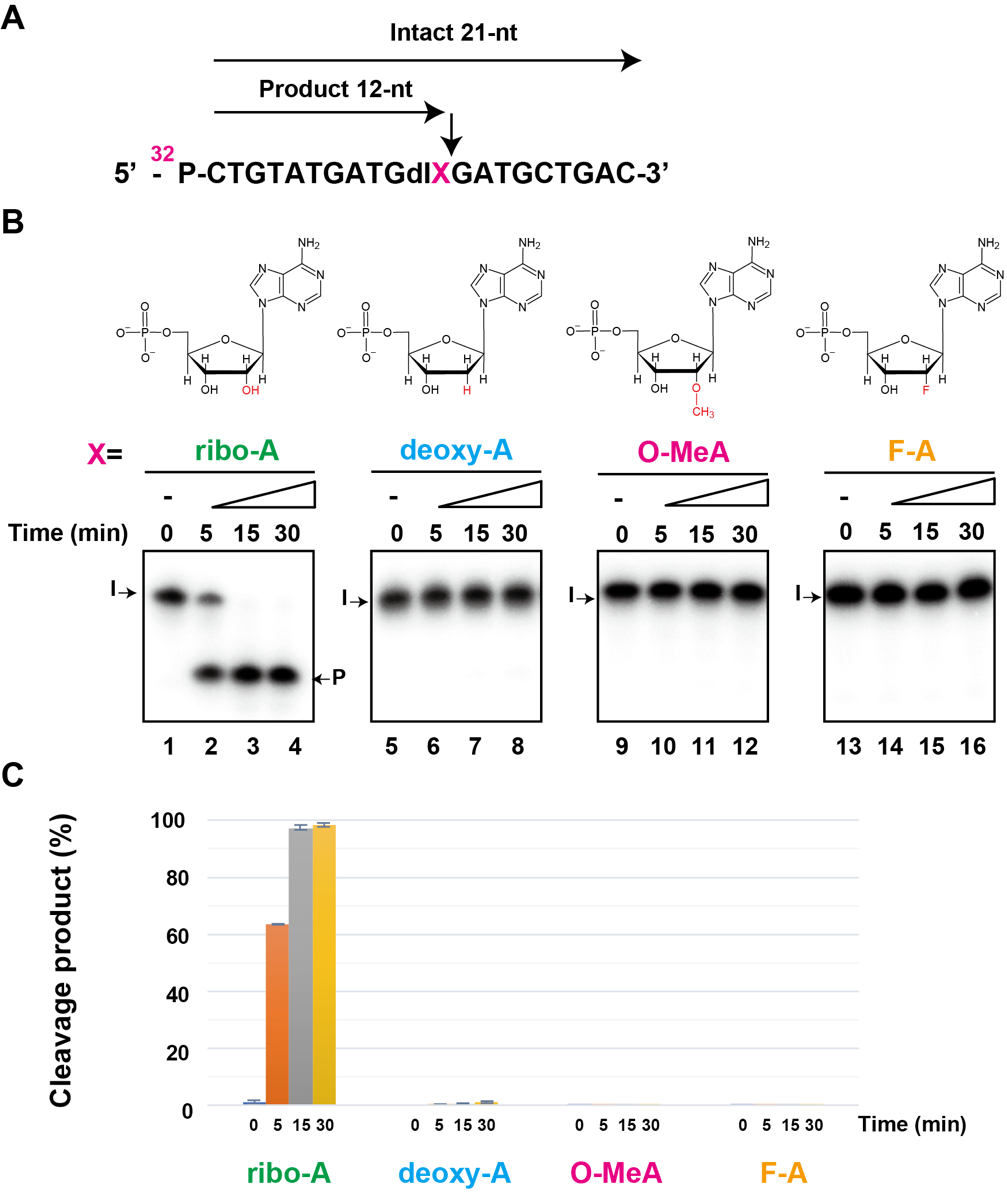
**

**Figure S2. hEndoⅤ cleaves deoxyinosine-containing RNA as a substrate**

(A) ^32^p-labeled 21nt single-stranded oligonucleotide containing deoxyinosine next to ribo-adenine, deoxy-adenine, 2′-O-Me-adenine, or 2′-F-adenine residues were used as substrates. Arrows indicate the position of the cleavage. Intact 21-nt; Intact oligonucleotide. Product 12-nt; cleavage products. (B) The ^32^P-labeled substrate containing deoxyinosine next to ribo-adenine residue (lanes 1–4), deoxy-adenine residue (lanes 5–8), 2′-O-Me-adenine residue (lanes 9–12) or 2′-F-adenine residue (lanes 13–16) were incubated with 5 nM hEndoⅤ at 37 ℃ for 0, 5, 15 and 30 min. The cleavage products were analyzed by denaturing 12.5% urea gel electrophoresis. The chemical structures of the riboadenine, deoxy-adenine, 2′-O-Me-adenine, and 2′-F-adenine residues are shown in the upper panel. The arrows mark the positions of intact oligonucleotide (I), and product (P) after hEndoV endonuclease action. (C) Graph showing the yield of cleavage products obtained from hEndoV. The products were quantified using a ImageJ Software. Error bars represent Standard Deviation (SD).Abbreviations: hEndoV, human endonuclease V; P, phosphorus; ribo-A, riboadenine; deoxy-A, deoxy-adenine; O-MeA, 2’-oxy-methyl-adenine; F-A, 2’-fluoroadenine


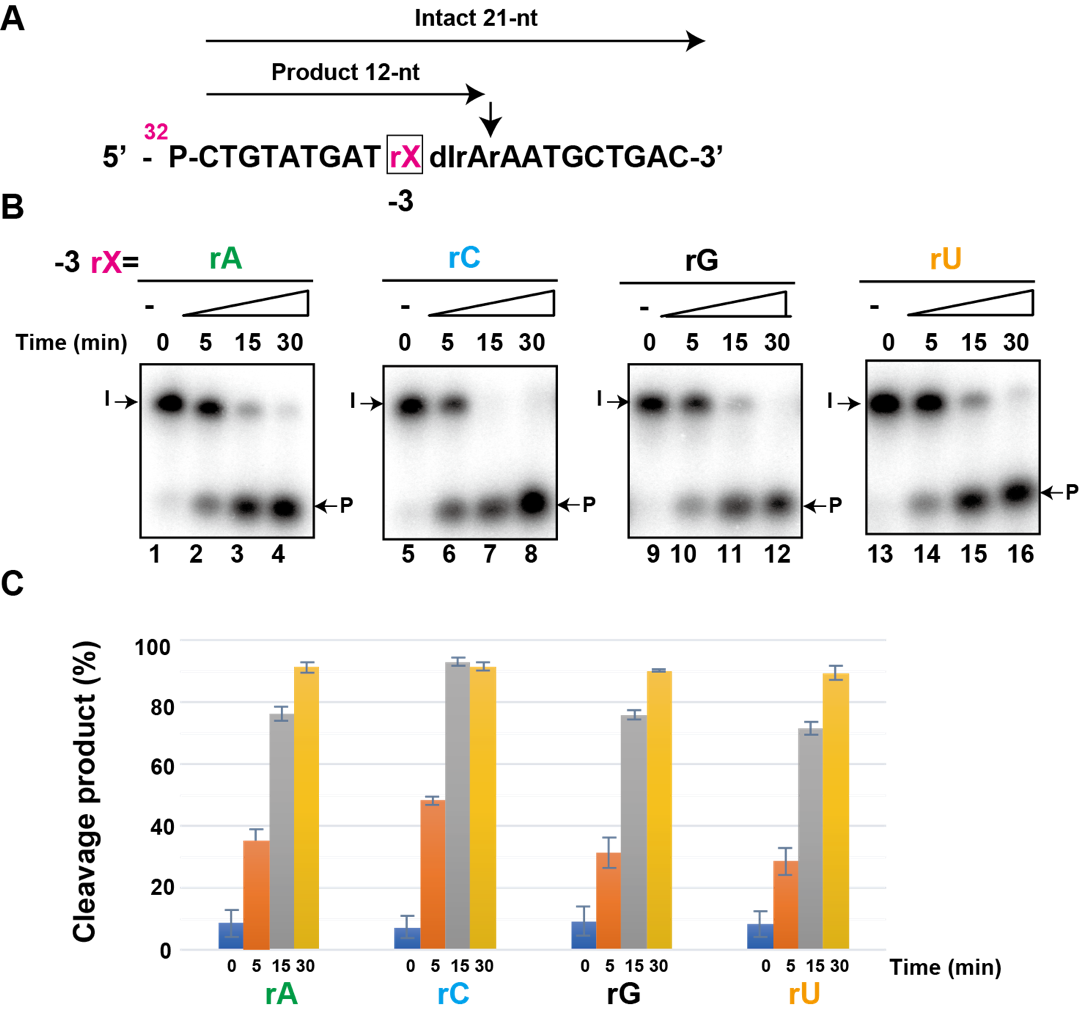


**Figure S3. The cleavage activity of hEndoV is unaffected by bases located on the 5′ end of deoxyinosine or 3′ end to the cleavage site**

(A) ^32^P-labeled substrates containing rX (rA, rC, rG, or rU) at the –3 positions are indicated. Arrows indicate the position of cleavage. Intact 21-nt; Intact oligonucleotide. Product 12-nt; cleavage products. (B) The ^32^P-labeled substrate containing rA (lanes 1–4), rC (lanes 5–8), rG (lanes 9–12), and rU (lanes 13–16) located at –3 positions were incubated with 5 nM hEndoⅤ at 37 ℃ for 0, 5, 15 and 30 min. The cleavage products were analyzed by denaturing 12.5% urea gel electrophoresis. The arrows mark the positions of intact oligonucleotide (I), and product (P) after hEndoV endonuclease action. (C) Graphs showing the yield of cleavage products obtained from hEndoV cleavage activity. The products were quantified using a ImageJ Software. Error bars represent Standard Deviation (SD). Abbreviations: hEndoV, human endonuclease V; P, phosphorus; rA, riboadenine; rC, ribocytosine; rG, riboguanine; ribo-U, ribouracil
